# Isolation and characterization of two phages infecting Acinetobacter baumannii and their effectiveness in treating BALB/c mice lung infections

**DOI:** 10.1099/jgv.0.002307

**Published:** 2026-08-12

**Keywords:** *Acinetobacter baumannii* infections, BALB/c mouse models, genome sequencing, phage cocktail, phages

## Abstract

*Acinetobacter baumannii* has raised significant concern due to its high rates of morbidity and mortality, coupled with its increasing resistance to multiple antibiotics including carbapenems. In this current study, two new lytic phages Srynta and Nolivar were isolated from sewage water, characterized *in vitro* and their therapeutic potential was assessed using a mouse model of pneumonia. *In vitro* results showed that both phages show efficient adsorption to the propagating host, have good lytic activity, short latent period and high burst size. Sequence analysis showed that Srynta and Nolivar phages have linear dsDNA genomes of 45,218 and 41,230 base pairs with G+C content of 38 mol% and 37.9 mol% and contain 88 and 83 coding sequences, respectively. Transmission electron microscopy showed the icosahedral capsid and contractile tail of the phages and genomic analysis predicted that both phages have myovirus morphology and belong to the class Caudoviricetes. Genomic analysis suggested the therapeutic safety of these phages, as they do not have bacterial virulence factors, antimicrobial resistance genes or lysogenic genes, suggesting that they are potential candidates for phage therapy. The *in vivo* study demonstrated a high survival rate in the phage cocktail treatment group compared to the control group. Our findings support the potential application of phage therapy as an alternative therapeutic treatment against lung infections caused by carbapenem-resistant *A. baumannii*.

**Impact Statement**

Phage treatment is confirmed and validated in this article as an antibiotic substitute. Newly isolated phages contribute to the growing repository of phages available for therapeutic use. Our study expands knowledge on the *in vitro* lytic activity and host range of these phages, while future research is needed to fully study their *in vivo* mechanism of action. DNA sequencing and genomic analysis further enrich our understanding of the genetic composition of phages and identify key genes responsible for virulence and replication. Computational analysis revealed the lytic nature of the newly isolated phages and broadened our understanding that they are safe for use as therapeutic agents. Animal trials further demonstrate the effectiveness of phages against drug-resistant bacteria. Our research on new lytic phages against *Acinetobacter baumannii* has a broad spectrum of interest and utility. It extends from addressing scientific inquiries in bacteriophage research to potentially impacting clinical practice, pharmaceutical development and public health strategies aimed at combating antibiotic resistance.

Positive outcomes in our research represent a significant step forward in validating the therapeutic potential of these phages. This stepwise validation paves the way for further preclinical and clinical studies.

## Data Summary

 All data and methods used in this study are provided in this article. Complete genomic sequences of A. baumannii phage Srynta and A. baumannii phage Nolivar were deposited in the NCBI GenBank database with accession numbers OR665530 and OR665531, respectively.

## Introduction

The proliferation of antibiotic-resistant bacteria stemming from the widespread application of broad-spectrum antibiotics has garnered significant attention. More recently, the emergence of pan drug-resistant pathogens, impervious to all antibiotics that are commercially available, poses substantial therapeutic challenges on a global scale [1]. *Acinetobacter baumannii*, a critical nosocomial pathogen, is accountable for diverse infections, including bacteraemia, urinary tract infections, pneumonia, wound infections and meningitis, particularly in immunocompromised patients in intensive care units. The rapid dissemination of multidrug-resistant (MDR) *A. baumannii*, an integral member of the ESKAPE pathogens, is a greater concern for medical facilities worldwide [2]. *A. baumannii*, named after Paul Baumann, serves as a primary representative of the *Acinetobacter* genus. This Gram-negative coccobacillus exhibits unique features, transitioning from a coccobacillus during the stationary phase to a rod-shaped morphology during the growth phase [3]. Historically, carbapenems have represented the pinnacle of potent and dependable β-lactam antibiotics for combating severe *A. baumannii* infections [4]. Carbapenem-resistant *A. baumannii* (CRAB) poses a substantial challenge to infection management due to its ability to resist treatment with carbapenem antibiotics. CRAB strains are typically resistant to nearly all available antibiotics. While colistin remains efficacious against most CRAB, its high toxicity designates it as a measure of last-resort treatment. As a result, therapeutic options for these infections are severely limited, and infections caused by CRAB are associated with substantial mortality [5]. The WHO bacterial priority pathogens list, 2024 (https://www.who.int/publications/i/item/9789240093461) has also declared CRAB as a critical pathogen, necessitating intensive research and development efforts for new therapeutic agents. Bacteriophages are viruses that exclusively target bacteria while leaving eukaryotic cells unharmed, offer promising alternatives to traditional antibiotics and have garnered significant attention over the last decade. This period has witnessed an influx of reports detailing the isolation and comprehensive characterization of diverse phages against *A. baumannii* [6]. In addition to bacteriophages themselves, their lytic enzymes, such as endolysins, have gained recognition as viable candidates for biocontrol purposes [7]. The foundation of phage therapy rests on the principle of viruses injecting their genetic material into host bacteria, inducing replication, subsequent cell lysis and eventual bacterial demise. While certain phages exhibit a high degree of host specificity, others display the capacity to infect a variety of host organisms [8]. Phages harnessed for therapeutic purposes bring about several advantages, including host specificity (which avoids disruption of normal flora and eukaryotic cells), rapid replication within bacterial hosts and precise elimination of target cells [9]. Notable instances of phages combating *A. baumannii* encompass both *in vitro* and *in vivo* scenarios. For instance, phage ISTD has shown efficacy against CRAB infections *in vitro* [10], whereas phage SH-Ab15519 has exhibited promise against CRAB infections both *in vitro* and *in vivo* [11]. In addition, the phage Βϕ-R2096 displayed anti-CRAB activity in *Galleria mellonella* larvae and in a mouse pneumonia model [12]. The combination of phages with antibiotics has been suggested to enhance therapeutic outcomes. The combination of phage vB_AbaM-KARL-1 and antibiotics yielded synergistic effects [13]. Moreover, the utilization of a five-phage cocktail in combination with antibiotics has exhibited a notable reduction in *A. baumannii* biofilms [14]. A growing body of evidence underscores the effectiveness of phage therapy in treating infections caused by drug-resistant bacteria. Instances of success encompass the recovery of patients afflicted with infections caused by antibiotic-resistant *A. baumannii* and non-tuberculous *Mycobacteria* through the application of phage therapy [15]. Notably, the Patterson case has garnered global attention as a recent example of effective phage therapy against a pan drug-resistant *A. baumannii* strain, resulting in substantial clinical improvement following a 2-day intravenous (i.v.) administration of tailored phage cocktail [16]. In the present study, we isolated two new *Acinetobacter* lytic phages, Srynta and Nolivar, from sewage water. Following meticulous characterization and sequence analysis, we proceeded to assess the therapeutic efficacy of phage cocktail against CRAB-induced pneumonia in a mouse model.

## Methods

### Bacterial strain

Six strains of *A. baumannii* were sourced from the Integrative Biology Lab (IBL-ASAB, NUST Islamabad) for the isolation of novel phages. These were originally isolated from sputum samples of lung infection patients from a local hospital in Islamabad. Antibiotic susceptibility profiling of all strains was carried out by disc diffusion method as described by Lysitsas *et al* [17]. Briefly, a single colony of each strain was mixed with normal saline and levelled with 0.5 McFarland standard solution turbidity. After vortexing, a standardized inoculum was evenly spread across Mueller–Hinton agar plates using a sterile swab. Antibiotic susceptibility discs were then kept on the agar, and plates were incubated at 37 °C for overnight. On the next day, the zone of inhibition was measured and analysed according to the antibiotic breakpoints mentioned by Clinical & Laboratory Standards Institute (https://clsi.org/).

### Isolation and purification of phages

Phage isolation was performed using the method as outlined by Sisakhtpour *et al*. [18]. Briefly, 40 ml of sewage sample was collected and centrifuged at 7,500 r.p.m. for 30 min to remove debris. The supernatant was then filtered through 0.22 µm sterile syringe filters to eliminate bacterial contaminants. Then, 1 ml of the filtered supernatant was mixed with 1 ml of an overnight culture of *A. baumannii* and incubated in 30 ml of Luria Bertani broth (OXOID CM1018) at 180 r.p.m. and 37 °C for 24 h. After incubation, 500 µl of chloroform (final concentration 1%) was added to the mixture to lyse bacterial cells. The suspension was centrifuged, and the supernatant was re-filtered using 0.22 µm syringe filters to obtain phage lysate. To confirm the presence of phages, a spot assay was performed [19]. Briefly, 100 µl of an exponentially growing *A. baumannii* 3–695 culture (OD600 ≈ 0.4–0.6) was spread on LB agar plates. Then, 10 µl of phage filtrate was spotted onto the bacterial lawn and incubated at 37 °C overnight. The appearance of lysis zones indicated the presence of phage activity, which was further confirmed and purified through plaque assay [20].

After overnight incubation at 37 °C, a single well-isolated plaque was picked and added to 30 ml of LB broth, along with 1 ml of an exponentially growing *A. baumannii* 3–695 culture (OD600 ≈ 0.4–0.6). After incubation for 24 h at 37 °C with shaking, 1% chloroform was added to lyse remaining bacterial cells. The supernatant was filtered through 0.22 µm filter, and the phage lysate was serially diluted. A total of 100 µl of diluted phage lysate was mixed with 3–5 ml of soft agar (0.6%) and overlaid onto LB agar plates. After overnight incubation at 37 °C, plaques were counted, and the process was repeated six to seven times until uniform plaque morphology was achieved. The phage quantification was determined using the formula described by Said *et al*. [21]

P.f.u. ml^−1^ = (plaques count ×1/dilution factor)/volume of phage plated (ml)

### Transmission electron microscopy

Negative staining was utilized to examine particulate suspensions [22]. A carbon-coated grid holding the adsorbed samples was exposed briefly to a droplet of negative stain (5% uranyl acetate), after which any surplus stain was removed. Subsequent to air drying, the grid underwent analysis using the JEOL JEM-1010 transmission electron microscope operating at 80 kV at the National Institute for Biotechnology and Genetic Engineering in Faisalabad.

### Thermal and pH stability assay

To evaluate phage stability under various environmental conditions, fresh phage lysates (1×10¹² p.f.u. ml^−1^) were mixed with an equal volume (1 ml) of LB broth. For thermal stability, mixtures were incubated for 1 h at different temperatures (4, 25, 37, 45, 55, 60, 65 and 70 °C). For pH stability, LB broth was adjusted to different pH levels [1, 3, 5, 7, 9, 11, 13] using 1 M HCl or 1M NaOH before autoclaving, and phage suspensions were incubated at 37 °C for 60 min. Following treatment, 100 µl of phage lysates were serially diluted in LB broth, and plaque assay was used to determine phage titre [23].

### Host range determination and efficiency of plating assay

The host spectrum of phages Srynta and Nolivar was assessed against six strains of *A. baumannii*, ten uropathic *Escherichia coli* strains, five strains of *Klebsiella pneumoniae*, four *Bacillus* strains and one *Staphylococcus aureus* strain. In this evaluation, 100 µl of a host culture in exponential growth phase (OD600≈0.4–0.6) was mixed with soft agar (0.6%) and then poured onto agar plates. Once it solidified at room temperature, 10 µl of the phage suspension (10^8^ p.f.u. ml^−1^) was spotted onto bacterial lawns and permitted to dry. Subsequently, plates were incubated at 37 °C overnight, and any observed zones of lysis were recorded [24]. The e.o.p. was determined to quantify phage infection efficiency across different *A. baumannii* strains. Spot-positive strains were further evaluated for e.o.p. with slight modifications to the previously described method [19]. Briefly, serial dilutions of phage lysate (10^8^ p.f.u. ml^−1^) were prepared and 100 µl of each dilution was mixed with 100 µl of exponentially growing bacterial strains (OD600≈0.4–0.6) followed by the double-layer agar overlay assay. Plates were incubated overnight at 37 °C, and plaques were counted to determine e.o.p. with following formula.

E.o.p. = (p.f.u. on test strain)/(p.f.u. on reference strain)

### Bacterial growth reduction assay

The reduction assay was conducted to assess the *in vitro* lytic efficacy of newly isolated phages. For this purpose, the host bacterium *A. baumannii* 3–695 was cultured in 30 ml LB medium at 37 °C until reaching an OD600 of 0.4. Subsequently, culture was mixed with the phages at m.o.i. values of 0.1, 1 and 10 [25]. Over the course of 6 h, 1 ml sample was collected at 1 h intervals during agitation at 37 °C. A spectrophotometer was employed to measure bacterial turbidity at OD600 nm in triplicates.

### Phage adsorption

An adsorption test was conducted to measure the quantity of phages adhering to *A. baumannii* 3–695 host cells within a defined timeframe. The host strain was cultivated in LB broth until it reached the exponential growth phase (OD600≈0.4–0.6). Subsequently, 1 ml of the culture was transferred into seven sterile microtubes, introducing the Srynta or Nolivar phage at a m.o.i. of 0.01. The mixed suspensions were incubated for varying durations (1, 3, 7, 10, 15 and 20 min) at 37 °C, and the suspensions were centrifuged to harvest bacterial cells with attached phages. The supernatant was utilized in a plaque assay to quantify the phages that were not adsorbed. By utilizing the initial phage concentration along with the unadsorbed phage concentration, the percentage of adsorbed phages was calculated using the formula as described by Geller *et al*. [26]

Percentage of adsorbed phages (%) = (1 − supernatant titre/stock titre) × 100

## One-step growth experiment and burst size

The life cycle of phages Srynta and Nolivar was investigated through an experiment of a one-step growth curve, unveiling the ‘burst size’, which quantifies the number of phages released from a single cell. First, *A. baumannii* 3–695 was inoculated in 15 ml of LB broth and allowed to incubate at 37 °C and 180 r.p.m. until it entered the exponential growth phase (OD600≈0.4–0.6). Following this, a 1 ml bacterial culture (1×10⁸ c.f.u. ml^−1^) was mixed with the phages at an m.o.i. of 0.01 (1×10⁶ p.f.u. ml^−1^). After a 7 min incubation at 37 °C to permit phage adsorption, the mixture was diluted 100-fold in LB broth to prevent interactions between unadsorbed phages and fresh cells. Over the ensuing 60 min, a 100 µl sample was collected every 10 min interval and phage titres were determined through a double-layer agar assay. Briefly, samples were serially diluted, and 100 µl of diluted phage lysate was mixed with 3–5 ml of soft agar (0.6%) and overlaid onto LB agar plates. After overnight incubation at 37 °C, plaques were counted. The burst size was computed by dividing the total number of released phages by the quantity of infected bacterial cells [10]

### Phage DNA extraction

The extraction of phage DNA was performed from purified phages using phenol/chloroform DNA extraction method as described by Liang *et al*. [27]. Initially, RNase A and DNase I were added to the phage suspension, reaching final concentrations of 1 µg ml^−1^ and 5 µg ml^−1^, respectively, to lyse bacterial RNA and DNA. After 1 h of incubation at 37 °C, Proteinase K (50 µg ml^−1^), 0.5 M EDTA and 10% SDS were added, followed by another 60 min of incubation at 56 °C. Subsequently, the suspension was subjected to extractions with equal volumes of chloroform, phenol/chloroform and phenol, followed by 10 min of centrifugation at 7,500 r.p.m.

DNA was precipitated by the addition of one-tenth volume of NaAc (3 mol l^−1^) and twofold volume of ethanol. This mixture was left at −20 °C for 30 min, followed by centrifugation at 10,000 r.p.m. at 4 °C for 30 min. The resulting nucleic acid was washed with 70% ethanol, dissolved in Tris Ethylenediaminetetraacetic acid buffer and the quality and concentration of the extracted DNA were measured using NanoDrop.

### Genome sequencing and bioinformatics analysis

Sequencing of phage DNA was conducted by Macrogen (Seoul, Republic of Korea) by employing the Nextera DNA XT Library preparation method along with the Illumina HiSeq platform, which generated paired end reads of 150 bp. The obtained raw reads underwent quality assessment using FastQC (Galaxy Version 0.74+galaxy1) [28]. To enhance data quality, Trimmomatic (Galaxy Version 0.36.6) was used to remove adapter sequences and low-quality reads [29]. The genome assembly was accomplished using Shovill (Galaxy Version 1.4.2+galaxy1), and statistics were evaluated using QUAST (http://cab.cc.spbu.ru/quast/).

The assembled genomes were annotated using the phage-specific pipeline Pharokka (Galaxy Version 1.3.2+galaxy0) and further validated through Prokka (Galaxy Version 1.14.6+galaxy1) [30] and RAST Toolkit (RASTtk version 2.0) (https://rast.nmpdr.org/) [31, 32]. Proteins annotated as hypothetical were further investigated using blastp searches against NCBI non-redundant protein database (https://blast.ncbi.nlm.nih.gov/Blast.cgi).

The linear genomic map of the phages was generated through clinker [33, 34]. TMHMM server (https://services.healthtech.dtu.dk/service.php?DeepTMHMM-1.0) was employed to predict the class of holin [35]. For predicting the genomic ends and mode of packaging of the Srynta and Nolivar phages, Phage Term (Galaxy Version 1.0.11) was employed [36]. PhageAI (https://phage.ai/) and BACPHLIP tool (Galaxy Version 1.0) were employed to predict the phage’s lifestyle. The therapeutic potential of the phages was evaluated using the PhageLeads platform (https://phageleads.dk/) [37]. This platform examines the phage genome for lysogeny relevant genes, antimicrobial resistance genes and genes linked with virulence. Virulence, resistance and toxin-associated genes in the Srynta and Nolivar phage genomes were also detected using Virulence Factor Database (http://www.mgc.ac.cn/VFs/), ResFinder (https://cge.food.dtu.dk/services/ResFinder/) [38] and RGI-CARD (https://card.mcmaster.ca/analyze/rgi) [39, 40]. Identification of potential genes encoding tRNA was carried out using tRNAscan-SE v.2.0 (http://trna.ucsc.edu/tRNAscan-SE/) [41, 42].

The DNA sequence of Srynta and Nolivar phages underwent similarity assessment against deposited sequences in the GenBank-NCBI database via blastn (https://blast.ncbi.nlm.nih.gov/Blast.cgi) [43]. Additionally, the JSpeciesWS tool (https://jspecies.ribohost.com/jspeciesws/) was employed to calculate the average nucleotide identity (ANIb) using blast+ [44]. The genomes were compared with the highest ANI phage genomes and visualized through ProgressiveMauve (https://darlinglab.org/mauve/mauve.html) [45].

### Phylogenetic analysis

Phylogenetic relationships among phage genomes were explored through a series of computational methods and tools. A tree of evolutionary relationship was generated through Virus Classification and Tree Building Online Resource (VICTOR) (https://victor.dsmz.de) by uploading blastn top-hit phage genomes similar to Srynta and Nolivar. The VICTOR was used for phylogenetic analysis and classification of prokaryotic viruses on the basis of whole genome sequences [46]. To perform pairwise nucleotide sequence comparisons, the phylogenomic GBDP tree was constructed using formula VICTOR_nt_tree_trimming_D6_FASTME. The balanced minimum evolution tree was inferred with the branch support of 100 pseudo-bootstrap via FASTME and taxon boundaries at family, genus and species level were estimated with the OPTSIL programme [47].

Additional phylogenetic trees were constructed through Molecular Evolutionary Genetics Analysis (mega 11.0) (https://www.megasoftware.net/) by using the neighbour-joining method with bootstrap value of 1,000 replicates [48, 49]. These trees were based on amino acid sequences of the major capsid protein and large subunit of terminase of Srynta, Nolivar and other relevant phages.

### Phage cocktail preparation

The preparation of the phage cocktail involved mixing equivalent volumes of each individual phage solution, all of which were at a concentration of 10^9^ p.f.u. ml^−1^. This resulting cocktail was stored at 4 °C until it was required for further use [50].

## *In vivo* phage treatment

**Fig. 1.**
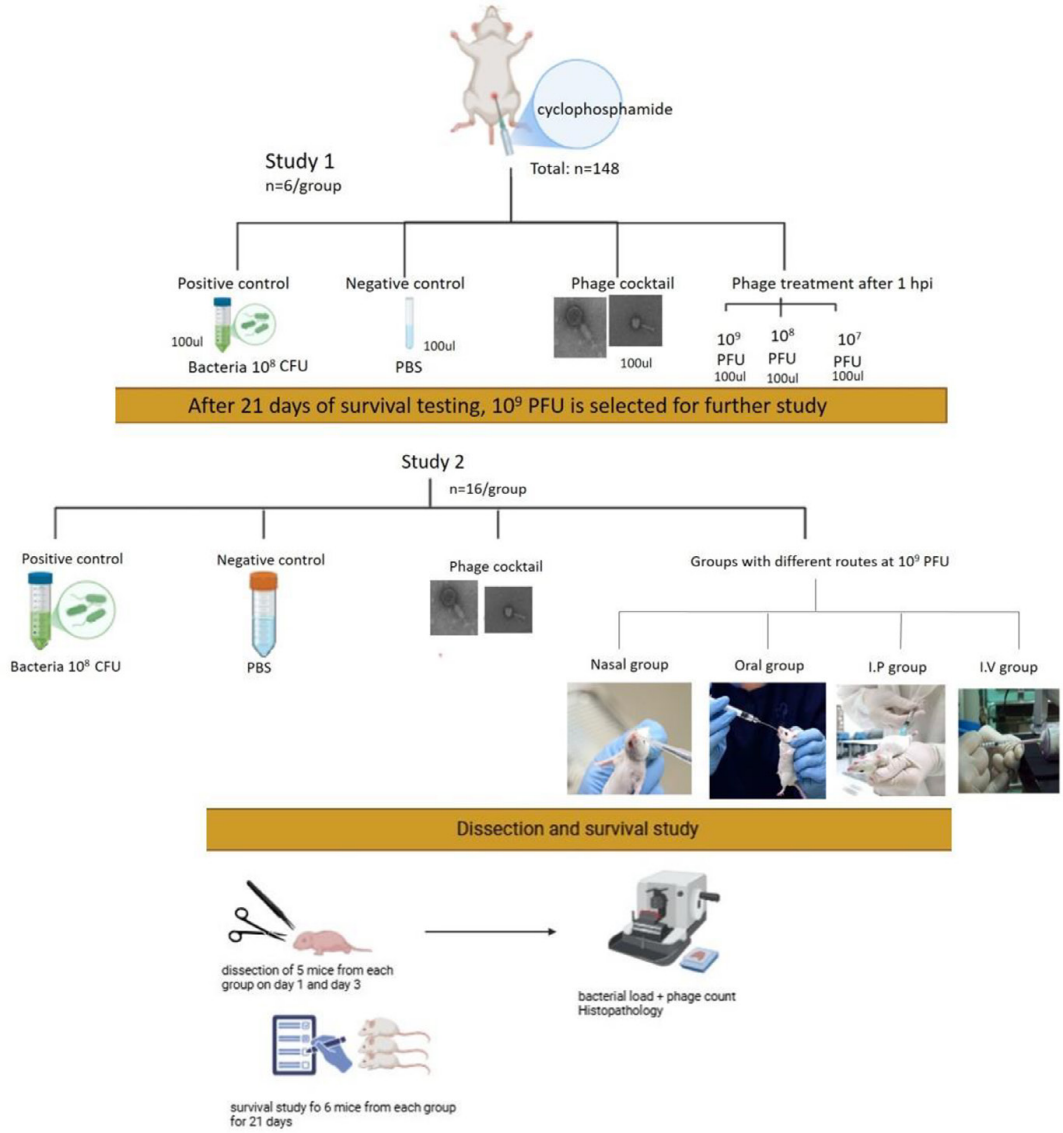
Schematic methodology of *in vivo* phage cocktail treatment. Two studies, depending on different dosage and administrative routes, were conducted to evaluate phage cocktail efficacy.

Two independent *in vivo* studies, as illustrated in Fig. 1, were conducted to evaluate the dose-dependent and route-specific therapeutic efficacy of the phage cocktail. A total of 148 female BALB/c mice (aged 7–8 weeks, weighing 20–25 g) were obtained from the Animal House Lab (ASAB-NUST Islamabad, Pakistan) and housed under controlled laboratory conditions. All animal handling and experimental protocols were conducted in accordance with institutional ethical guidelines. Two different doses of cyclophosphamide (150 mg kg^−1^) were administered to mice on days 1 and 4 before bacterial injection. The phage cocktail was formulated by adding an equal volume of both phages at the same concentration (p.f.u. ml^−1^). In study 1, three phage cocktail treatment groups with different m.o.i. values, along with three control groups (bacteria only, PBS only and phage cocktail only; *n*=6 each), were evaluated to determine the best m.o.i. [11]. Mice received 100 µl intraperitoneal (i.p.) bacterial inoculation at 10^8^ c.f.u. ml^−1^ followed by 1 h post-infection (hpi) intranasal inoculation of 100 µl phage cocktail at concentrations of 10^9^, 10^8^ and 10^7^ p.f.u. ml^−1^. Survival, mortality and clinical signs were observed for the following 21 days of the study. After selecting the optimal m.o.i., study 2 evaluated phage cocktail efficacy via nasal, oral, i.p. and i.v. routes alongside three control groups (*n*=16/group). A total of 100 µl of PBS and exponentially (OD600≈0.4–0.6) growing bacteria at a concentration of 10^8^ c.f.u. ml^−1^ were injected intraperitoneally into the mice of buffer-only and bacteria-only groups, respectively. In the phage cocktail treatment groups, 100 µl of *A.baumannii* 3–695 (at a concentration of 10^8^ c.f.u. ml^−1^) was injected through an i.p. route and at 1 hpi 100 µl phage cocktail at m.o.i. 10 (10^9^ p.f.u. ml^−1^) was injected through four different routes (i.p, i.v, oral and nasal). In another group, only phage cocktail at a concentration of 10^9^ p.f.u. ml^−1^ was also administered intranasally. From each group, five mice were sacrificed on days 1 and 3 for bacterial/phage load and histopathology, while the remaining six were monitored for 21 days for survival and clinical signs [12].

### Bacterial burden and phage titer

On days 1 and 3 post-infection, mice were euthanized and lungs from each group were aseptically harvested, homogenized in 500 µl sterile PBS and centrifuged to remove tissue debris. For bacterial load estimation, lung homogenates (100 µl) were serially diluted in PBS and spread on LB agar plates. After overnight incubation at 37 °C, colonies were manually counted to determine c.f.u. lung^−1^. In the meantime, 100 µl of lung homogenate was filtered using 0.22 µm syringe filters and serially diluted in PBS and plated using the double agar overlay method. After overnight incubation, plaques were counted and titre was calculated as p.f.u. lung^−1^ [51].

### Histology of mouse lungs

On days 1 and 3 post-infection, mice were euthanized and lungs from each group were aseptically harvested in 10% formalin. Commercial Haematoxylin and Eosin (H and E) staining was conducted, and slides were examined using an OPTIKA microscope [52].

### Statistical analysis

GraphPad Prism 9.5.1.733 software and R programming were used for statistical calculations. Survival curves between groups were compared using the log-rank (Mantel-Cox) test. To compare statistical calculations of bacterial load and phage titre across different treatment groups, one-way ANOVA with Tukey’s multiple comparison test was performed. Every test was performed in triplicates and *P*<0.0001.

## Results

### Bacterial strains and isolation of phages

**Fig. 2.**
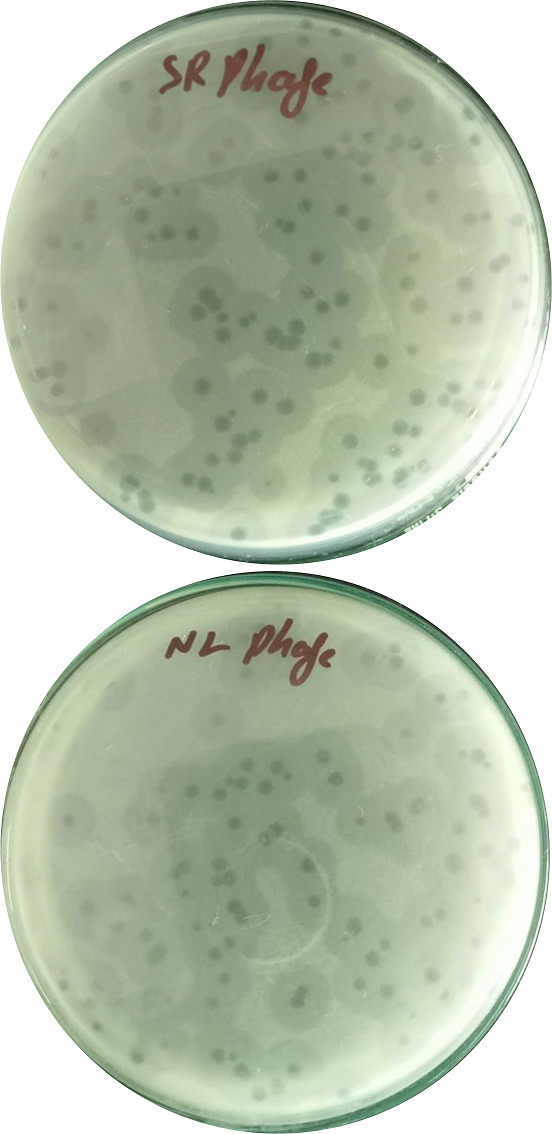
Plaque morphology of Srynta and Nolivar phages. *A. baumannii* phages exhibited transparent, spherical plaques encircled by halos. SR: Srynta, NL: Nolivar.

All bacterial strains used in this study were carbapenem-resistant, tetracycline- and colistin-sensitive (Table 1). Two new closely related *A. baumannii* phages, Srynta and Nolivar, were isolated from sewage samples collected from Nala Lai, Rawalpindi/Islamabad, Pakistan. Phages exhibited similar plaque morphologies and formed clear, round plaques with noticeable halos around them (Fig. 2). Initially, the phage titre was ~10^12^ p.f.u. ml^−1^ and was kept at 4 °C for further use. However, we observed that with prolonged storage, the titres gradually declined to 10¹⁰–10⁹ p.f.u. ml^−1^, aligning with the ranges reported in the literature.

**Table 1.** Antibiotic susceptibility profiling of *A. baumannii* strains. Zones were measured in mm. Strains were identified as sensitive (S) or resistant (R) according to published CLSI breakpoints

Serial no.	Bacterial strain	Colistin (CT) (10 µg)	Meropenem (MEM) (10 µg)	Imipenem (IPM) (10 µg)	Tetracycline (TE) (30 µg)	Ceftazidime (CAZ) (30 µg)	Amikacin (AK) (30 µg)	Levofloxacin (LEV) (5 µg)
1	*A. baumannii* 4–311	≤ 16 S	≤ 12 R	0 R	≤ 27 S	0 R	0 R	0 R
2	*A. baumannii* 7–824	≤ 15 S	≤ 14 R	0 R	≤ 26 S	0 R	0 R	0 R
3	*A. baumannii* 1–181	≤ 17 S	≤ 14 R	0 R	≤ 25 S	0 R	0 R	0 R
4	*A. baumannii* 3–695	≤ 15 S	≤ 12 R	0 R	≤ 22 S	0 R	≤ 8 R	0 R
5	*A. baumannii* 2–735	≤ 14 S	≤ 14 R	0 R	≤ 17 S	0 R	0 R	≤ 11 R
6	*A. baumannii* 8–924	≤ 15 S	≤ 12 R	0 R	≤ 22 S	0 R	≤ 9 R	0 R

### Transmission electron microscopy

**Fig. 3.**
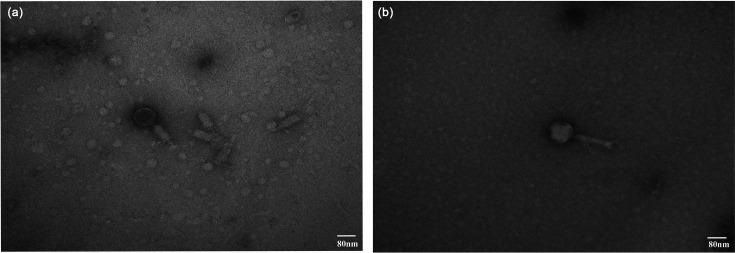
TEM morphology of Srynta (**a**) and Nolivar (**b**) phages. The scale bar represents 80 nm.

Transmission electron microscopy (TEM) images of phages Srynta and Nolivar revealed icosahedral heads and contractile tails, indicating their classification within the class Caudoviricetes. The head diameter of Srynta was 87±10 nm (*n*=4), whereas Nolivar had a diameter of 91±5 nm (*n*=4). The contractile tail dimensions were 143×30 nm for Srynta phage and 163×18 nm for Nolivar phage, measured through ImageJ (Fig. 3).

### Thermal and pH stability assay

**Fig. 4.**
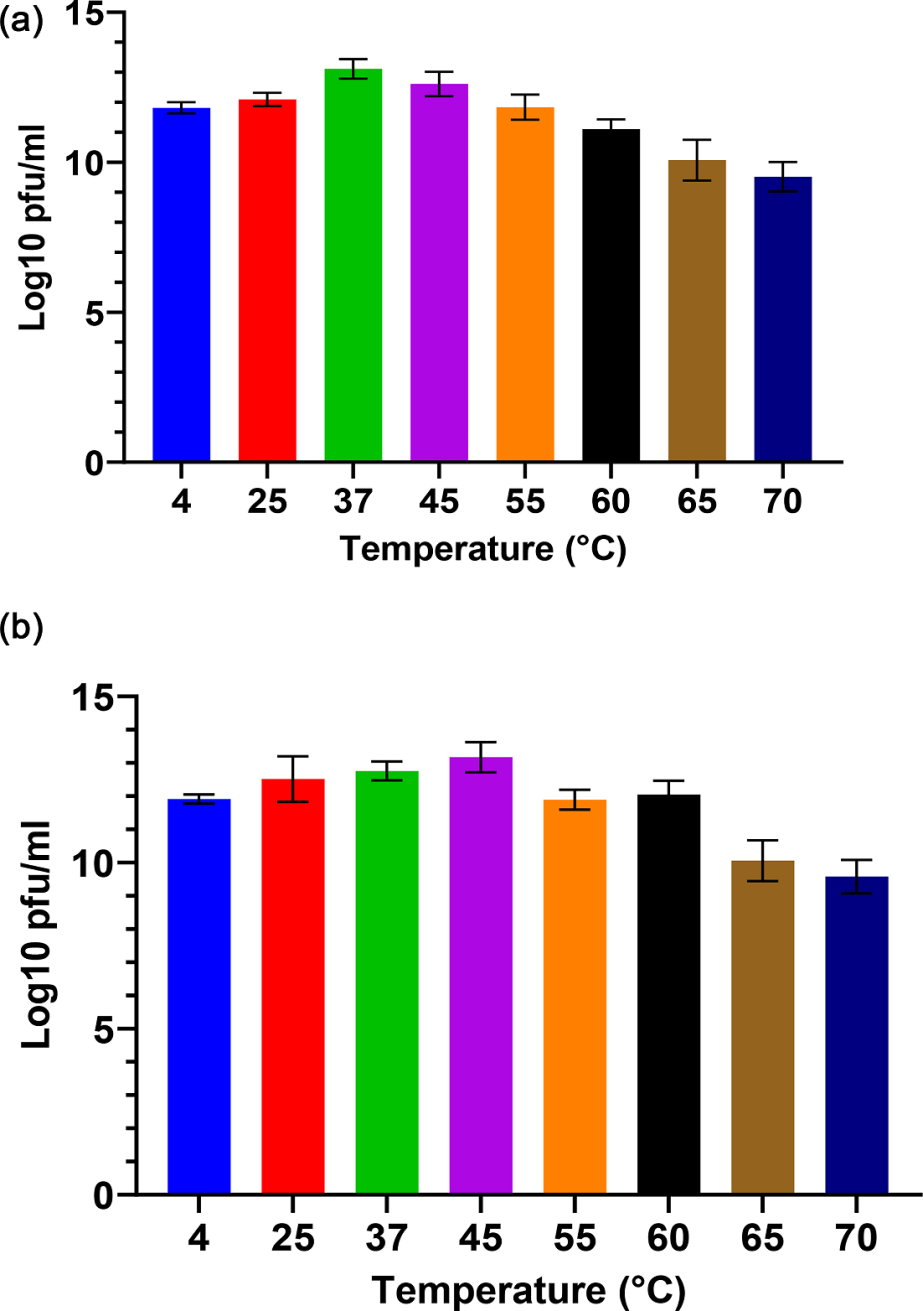
Thermal stability of *A. baumannii* phages Srynta (**a**) and Nolivar (**b**) at various temperatures. The data shown are the mean±sd, and it was derived from three separate findings.

**Fig. 5.**
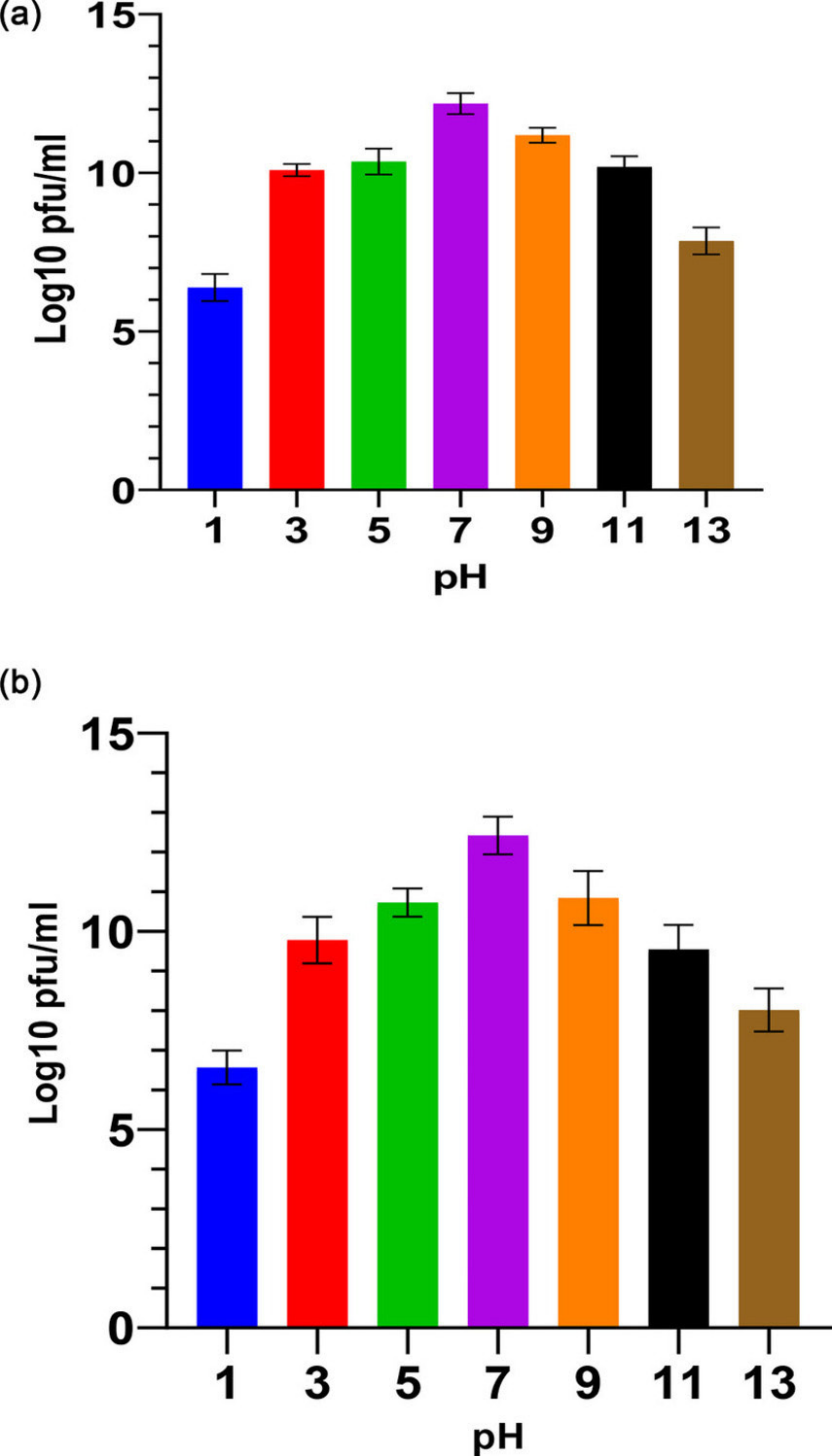
pH stability of phage Srynta (**a**) and Nolivar (**b**). The mean±sd values of the data are given for three replicates.

Thermal stability tests indicated that both phages remained highly stable within the temperature range of 4–60 °C (Fig. 4). Using 37 °C as a reference point for Srynta, log_10_ p.f.u. ml^−1^ reductions of 2.27 at 65 °C and 3.60 at 70 °C were observed. For Nolivar, the reduction from the peak at 45 °C was log_10_ p.f.u. ml^−1^ 2.47 and 3.18 at 65 and 70 °C, respectively. For both phages, a reduction of only 0.2–0.5 log_10_ p.f.u. ml^−1^ compared to their optimal titres was observed at 4 °C, which still shows their excellent stability at low temperatures. Additionally, both phages demonstrated high stability across the pH range of 3–11, with maximum at pH 7 (Fig. 5). Compared to this, Srynta showed a reduction of 5.80 log_10_ p.f.u. ml^−1^ at pH 1 and 2.10 log_10_ at pH 3, whereas Nolivar exhibited reductions of 5.96 and 2.51 log_10_ p.f.u. ml^−1^ at pH 1 and 3, respectively. Both phages were found to be more stable in basic conditions.

### Host range determination and efficiency of plating assay

Spot test and e.o.p. assay results revealed that both phages lysed five out of six tested *A. baumannii* strains, with no lytic activity against all other tested strains of *Bacillus, E. coli*, *K. pneumoniae* and *S. aureus* (Table 2). Based on its high susceptibility and strong e.o.p., *A. baumannii* 3–695 was selected for further characterization and *in vivo* studies.

**Table 2.** Host range analyses and e.o.p. values of Srynta and Nolivar phages. ‘+’ sign denotes the presence of a clear spot, while ‘-’ sign represents the absence of a lysis zone

Serial no.	Bacterial strain	Lysis by Srynta	Lysis by Nolivar	E.o.p. (Srynta)	E.o.p. (Nolivar)
1	*A. baumannii 3–695*	+	+	1.0000	1.0000
2	*A. baumannii 2–735*	+	+	0.6552	0.5600
3	*A. baumannii 4–311*	+	+	0.1241	0.1296
4	*A. baumannii 1–181*	–	–	0.0000	0.0000
5	*A. baumannii 8–924*	+	+	0.0724	0.1264
6	*A. baumannii 7–824*	+	+	0.0931	0.1480

### Bacterial growth reduction assay

**Fig. 6.**
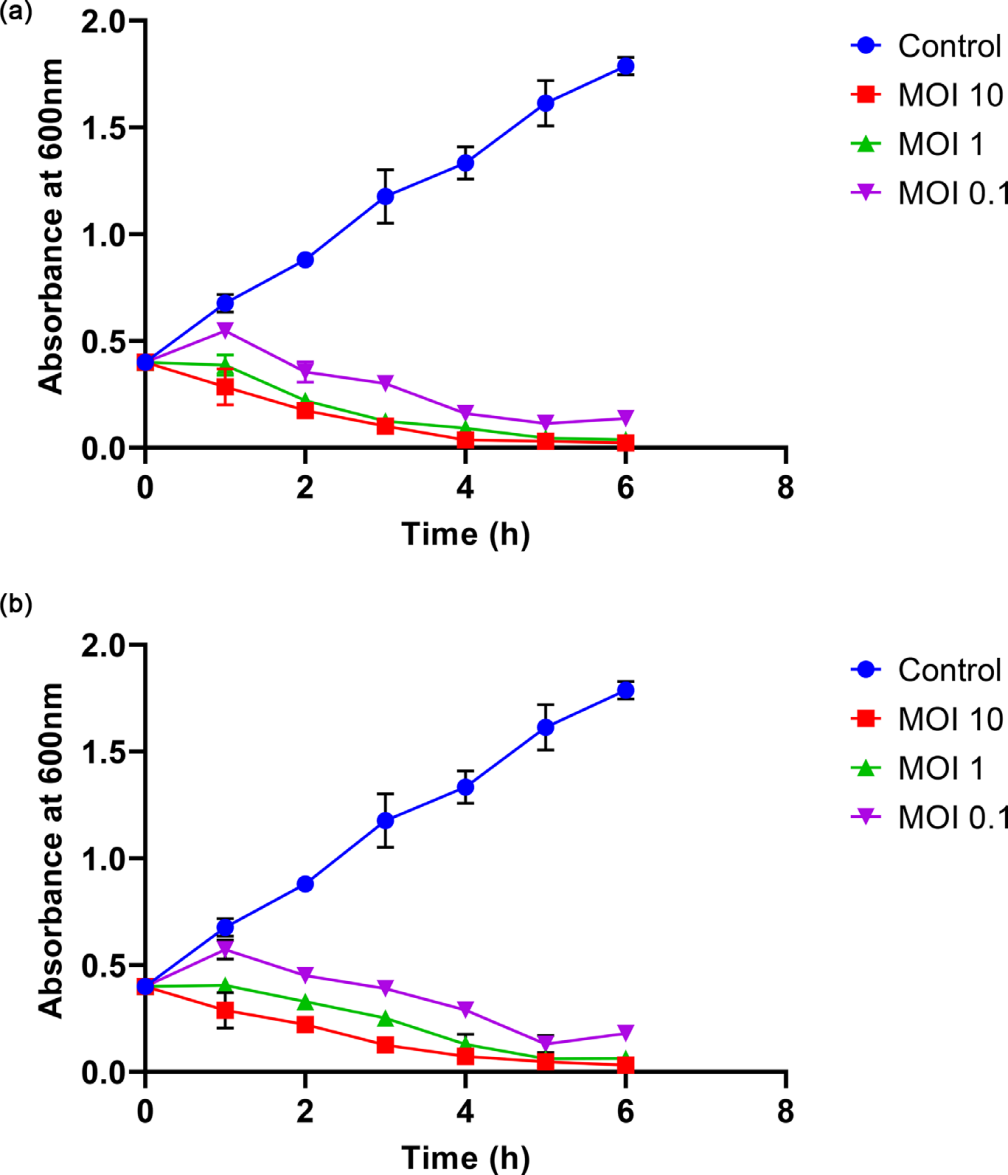
Lytic kinetics of phages Srynta (a) and Nolivar (**b**) at m.o.i. values of 0.1, 1 and 10 against *A. baumannii* 3–695. Both phages reduced bacterial growth to 6 h even at m.o.i. 0.01. The mean±sd is used in data.

The concentration-dependent lytic activity of phages Srynta and Nolivar against *A. baumannii* 3–695 was assessed *in vitro*. Both phages effectively lysed and reduced bacterial growth up to 6 h even at low m.o.i. (0.01), as compared to control (Fig. 6).

### Phage adsorption

**Fig. 7.**
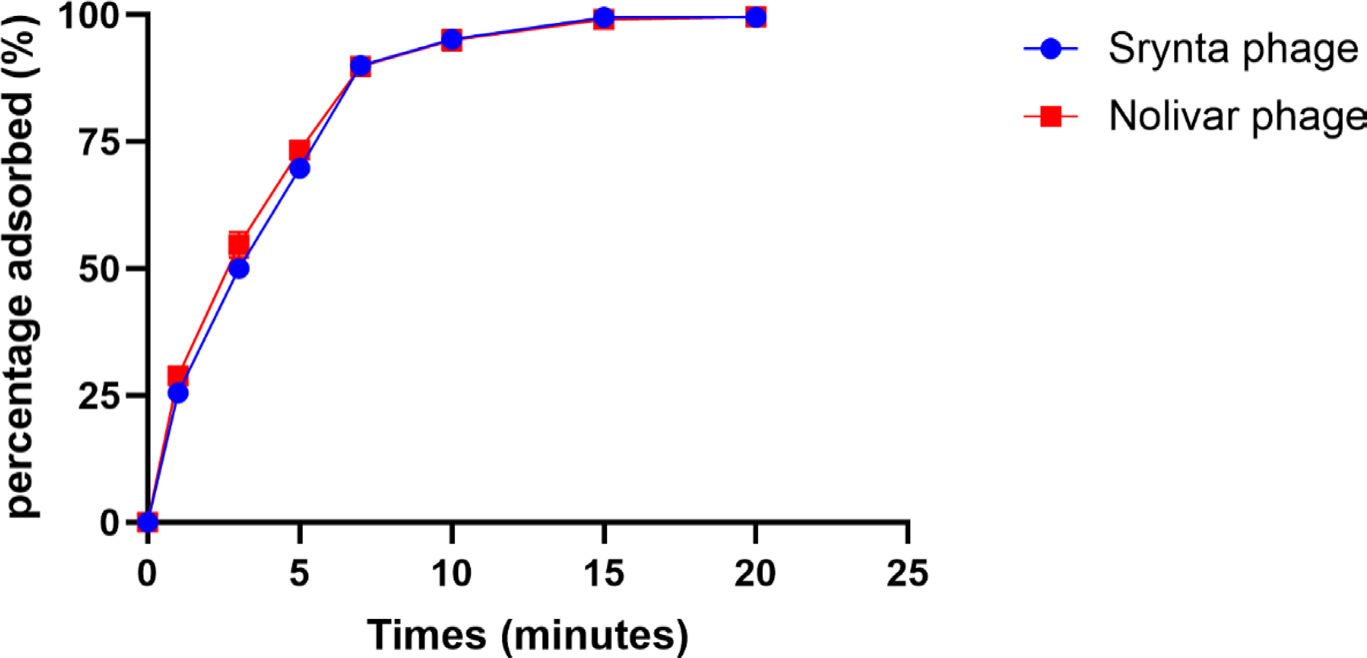
Adsorption kinetics of *A. baumannii* phages Srynta and Nolivar. The graph presents mean values from three independent experiments.

Phage adsorption experiments demonstrated that both Srynta and Nolivar phages can rapidly be adsorbed to their host cells. Both Nolivar and Srynta phages achieved 90, 95 and 100% adsorption within 7, 10 and 15–20 min, respectively (Fig. 7).

### One-step growth experiment and burst size

**Fig. 8.**
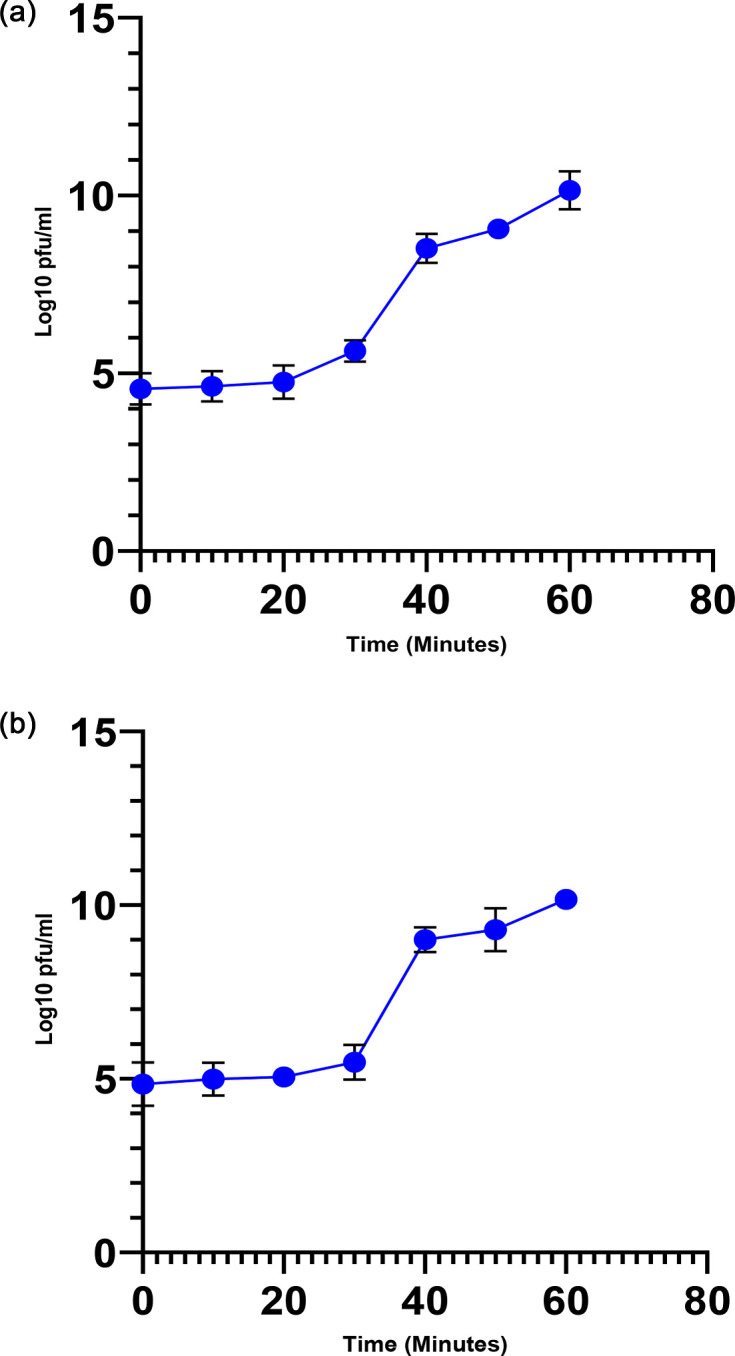
One step growth curve of phages Srynta (**a**) and Nolivar (**b**). Srynta and Nolivar have 30 min latent period and burst sizes of 100 and 81 p.f.u. infected cell^−1^, respectively. Data represents mean±sd from three repeats.

To comprehend the virus life cycle, one-step growth curves were constructed. These curves track the virus infection cycle and estimate burst size. Analysis of one-step growth curves for Srynta and Nolivar phages revealed a latent period of 30 min and burst size of 100 and 81 p.f.u. infected cell^−1^, respectively (Fig. 8).

### Genome sequencing and bioinformatics analysis

The genomes of Srynta and Nolivar were 45,218 bp and 41,230 bp in length, respectively, with G+C contents of 38.0 mol% and 37.9 mol%. Sequencing coverage reached 150.8× for Srynta and 83× for Nolivar. Genome annotation predicted 88 coding sequences (CDSs) in Srynta and 83 CDSs in Nolivar. Putative functions could be assigned to 39 CDSs in Srynta and 36 CDSs in Nolivar, whereas the remaining CDSs encoded hypothetical proteins.

**Fig. 9.**
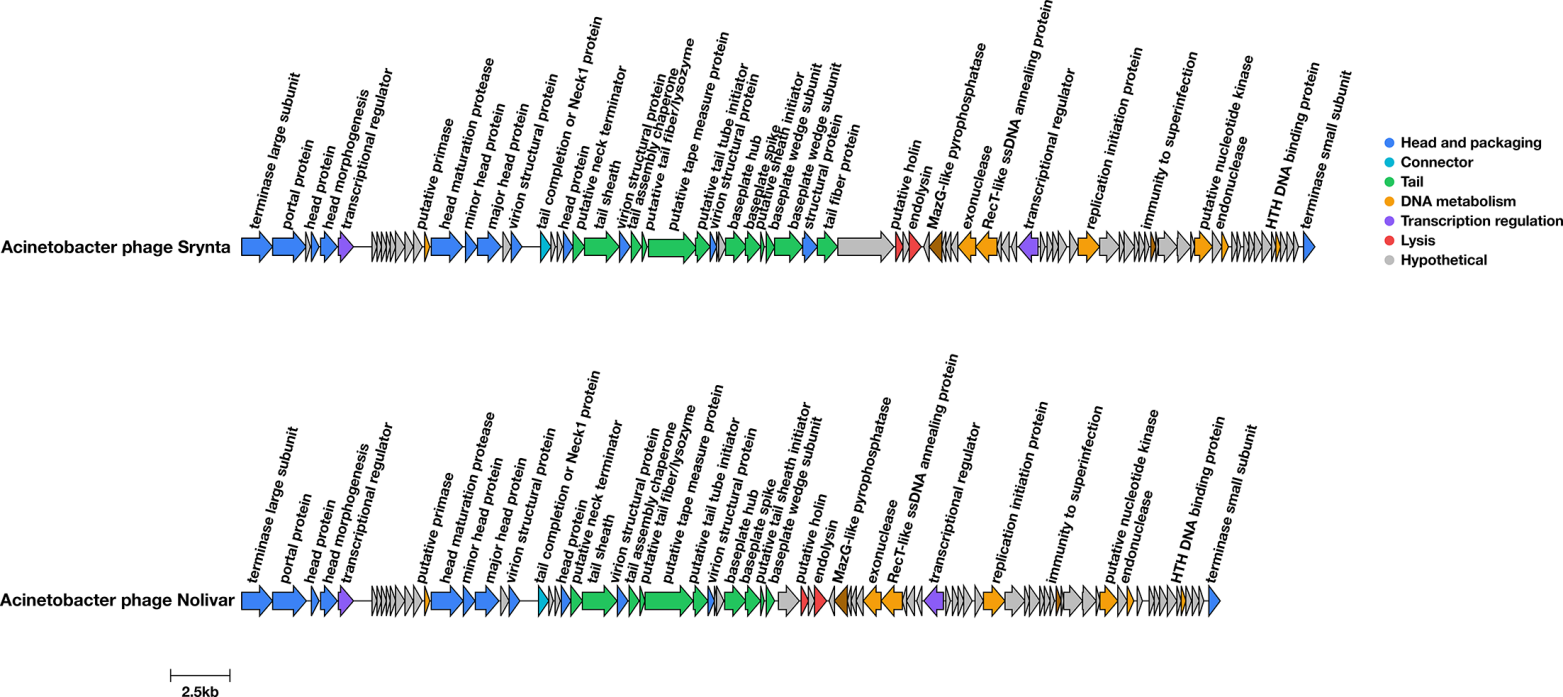
Linear genomic map of phages Srynta (**a**) and Nolivar (**b**). Various functional modules are represented by different colours.

**Fig. 10.**
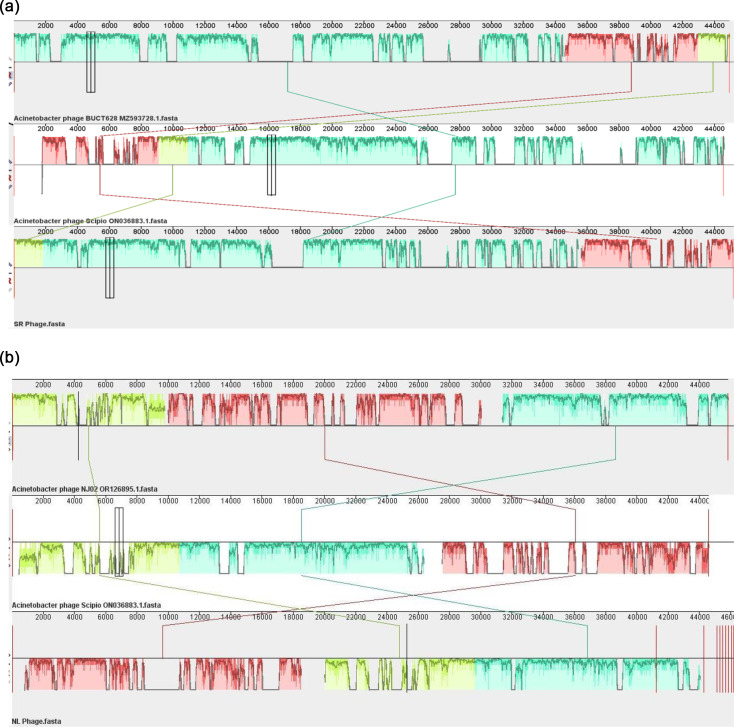
Comparative genomic map of Phage Srynta (**a**), Nolivar (**b**) and their relevant phages. The Srynta phage is compared with Scipio ON036883.1, and *Acinetobacter* phage BUCT628 MZ593728.1 as well as Nolivar phage is compared with *Acinetobacter* phages NJ02 OR126895.1 and Scipio ON036883.1. The colours used to signify genomic regions that are homologous are the same.

Comparative analysis of genome organization revealed a highly conserved gene arrangement between the two phages (Fig. 9). Based on predicted functions, annotated genes were grouped into modules associated with head and packaging, connector, tail proteins, DNA metabolism and replication, transcriptional regulation and host lysis.

blastn results showed that the phages Srynta and Nolivar are similar to *A. baumannii* phage vB_AbaM_GW418IMT19 with 95.88% identity and 68 and 69% coverage, respectively. The ANIb values obtained from jspeciesWS underscored their relatedness to other phages, although none surpassed the 95% threshold (Fig. 10). A 95% ANIb threshold is commonly used as a species demarcation in prokaryotic genomics [53]. This cutoff has been widely adopted in microbial taxonomy as it reflects the genomic similarity level at which two strains are typically considered to belong to the same species [54].

Furthermore, Phage Term indicated that both Srynta and Nolivar phages possessed terminally redundant genomes with partially circular permutations, indicative of a headful packaging mode. Phage AI, BACPHLIP and PhageLeads exhibited the virulent lifestyle of both *Acinetobacter* phages Srynta and Nolivar. No tRNA-encoding gene, AMR gene, bacterial virulence factors and lysogeny-associated genes were predicted in genomic analyses. Collectively, comprehensive genomic analyses provide robust evidence supporting the safety and potential therapeutic applications of phages Srynta and Nolivar.

### Phylogenetic analysis

**Fig. 11.**
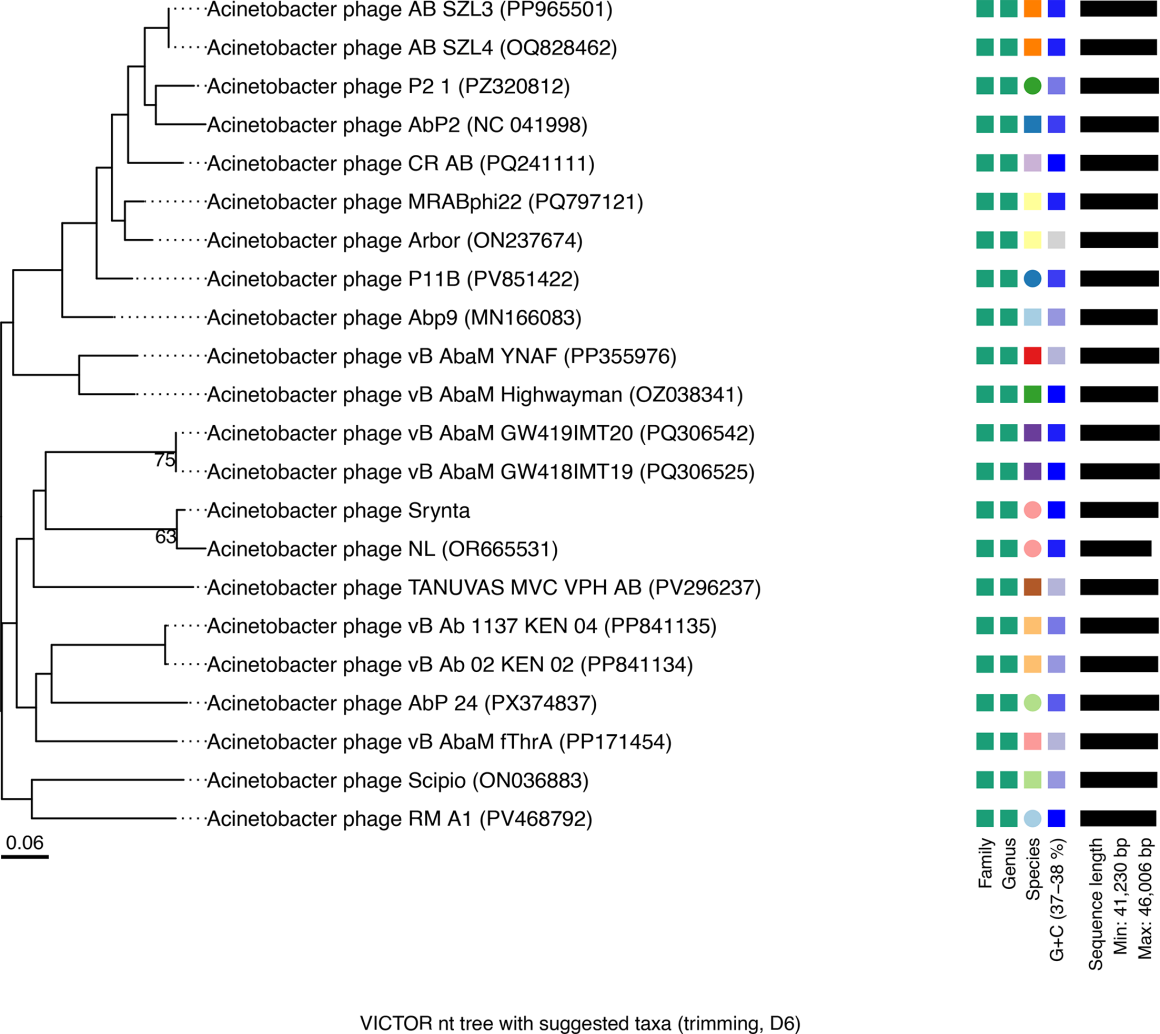
A phylogenetic tree based on the VICTOR genome was constructed by comparing the nucleotide sequences of Srynta, Nolivar and other closely related phages. The number of substitutions per site is shown by the scale bar. Bootstrap support values are represented by numbers at each branch point.

**Fig. 12.**
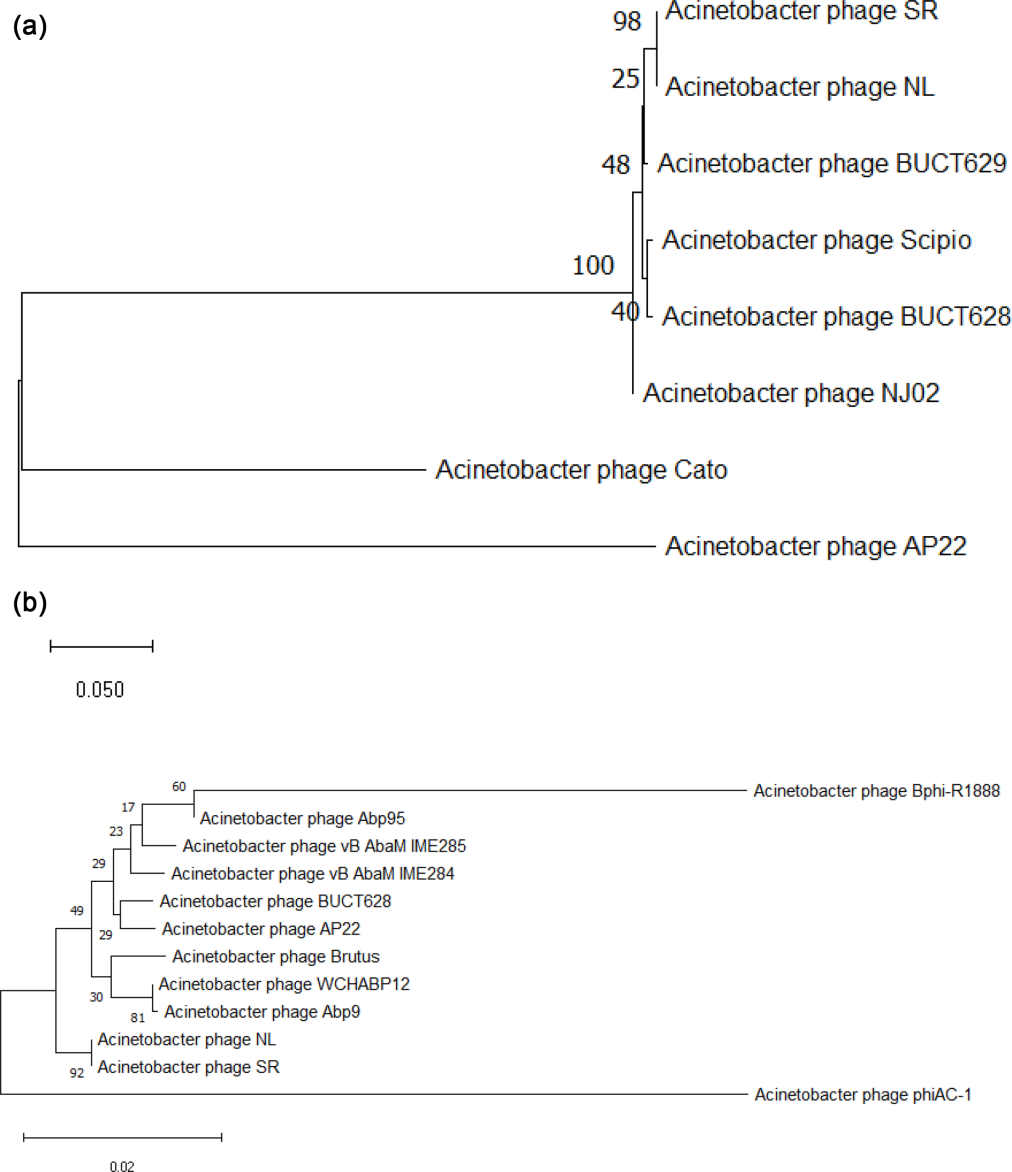
Evolutionary trees based on protein sequences of the main primary capsid protein (**a**) and large subunit of terminase (**b**). The number of substitutions per site is indicated by scale bar. Bootstrap support values are represented by numbers at each branch point.

Phylogenetic analysis, utilizing various methods, provides further insight into the relationship between already reported *Acinetobacter* phages with Srynta and Nolivar. VICTOR clustering positioned phages Srynta and Nolivar alongside the top blastn hits, encompassing 22 phages within the Caudoviricetes class and the genus *Obolenskvirus*. It was notable that both phages represented distinct strains among this grouping. This suggested their classification within the class Caudoviricetes and the genus *Obolenskvirus*. Furthermore, VICTOR’s construction revealed that the newly isolated *Acinetobacter* phages Srynta and Nolivar were closely related to each other as compared to previously reported phages (Fig. 11). Phylogenetic trees based on amino acid sequence of major capsid protein and terminase large subunit revealed the close proximity of phages Srynta and Nolivar (Fig. 12).

### *in vivo* phage treatment

**Fig. 13.**
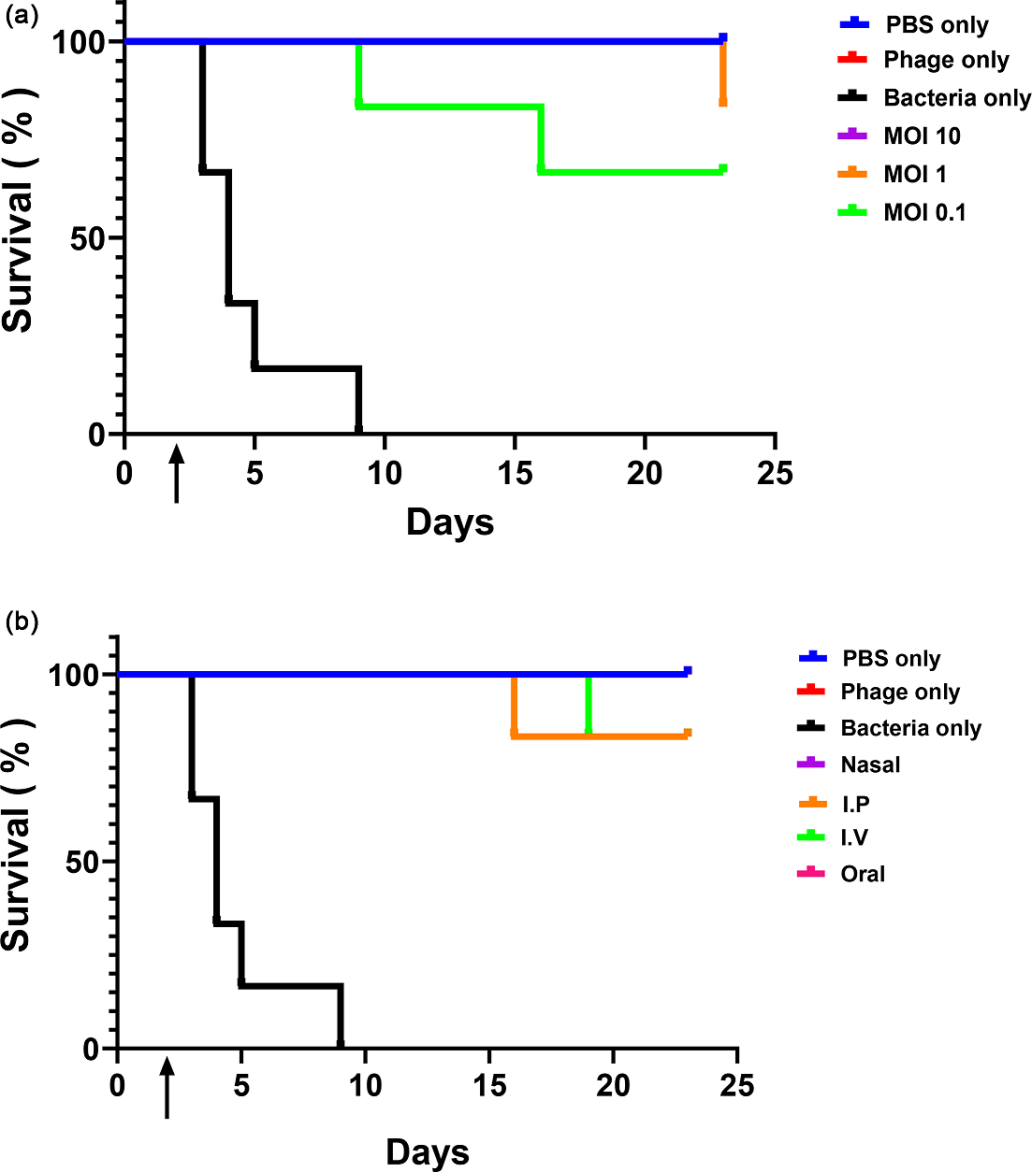
(a) *In vivo* dose-dependent therapeutic evaluation of phage cocktail against *A. baumannii* 3–695. A total of 100 µl of *A*. *baumannii* 3–695 was first administered intraperitoneally on day 2, and then at 1 hpi, they were given 100 µl intranasal phage cocktail at varying m.o.i. values (10, 1, 0.1). *P*<0.0001. (b) Survival kinetics of mice treated with phage cocktail through different administrative routes. The mice were inoculated with *A. baumannii* 3–695 (108 c.f.u. ml^−1^) intraperitoneally on day 2 and then given a phage cocktail (109 p.f.u. ml^−1^) by one of four modes of administration (nasal, oral, i.p. and i.v.) at 1 hpi. *P* < 0.0001.

Our study aimed to assess the impact of a cocktail of *A. baumannii* phages Srynta and Nolivar as a potential antibacterial treatment in a mouse model of pneumonia. The experimental model involved inducing lung infections in mice through i.p. inoculation of *A. baumannii* 3–695. To assess the therapeutic effect of the phage cocktail, various doses were administered intranasally 1 h after infection. Our findings revealed that treatment with phages at m.o.i. values of 10, 1 or 0.1 significantly improved the survival rates of infected mice compared to the non-phage-treated control group. In contrast, the group infected with bacteria only experienced a 0% survival rate by day 7, while the mice receiving phage treatment exhibited substantially higher survival rates by day 21: m.o.i. 10 (100%), m.o.i. 1 (83%) and m.o.i. 0.1 (66%) (Fig. 13). Notably, neither the phage-only group nor the control group treated with the buffer experienced any fatalities. It is worth noting that among the phage cocktail treatment groups, the m.o.i. of 10 led to milder clinical symptoms, such as piloerection, lethargy and reduced activity, compared to the other two dosages with moderate-to-severe symptoms of ruffled fur, reduced mobility, hunched posture and transient lethargy. This observation prompted us to adopt m.o.i. 10 as the standard dose for further experiments.

Survival rate analysis underscored the effectiveness of phage treatment through different administration routes compared to the bacteria-only group. Over the course of our 3-week survival study, the nasal and oral routes of the phage cocktail administration demonstrated 100% survival rates, whereas intravascular and i.p. routes yielded an 83% survival rate. Notably, one mouse each from the i.p. and intravascular groups succumbed at 14th and 17th days post-infection, respectively (Fig. 13b).

### Bacterial burden and phage titer

**Fig. 14.**
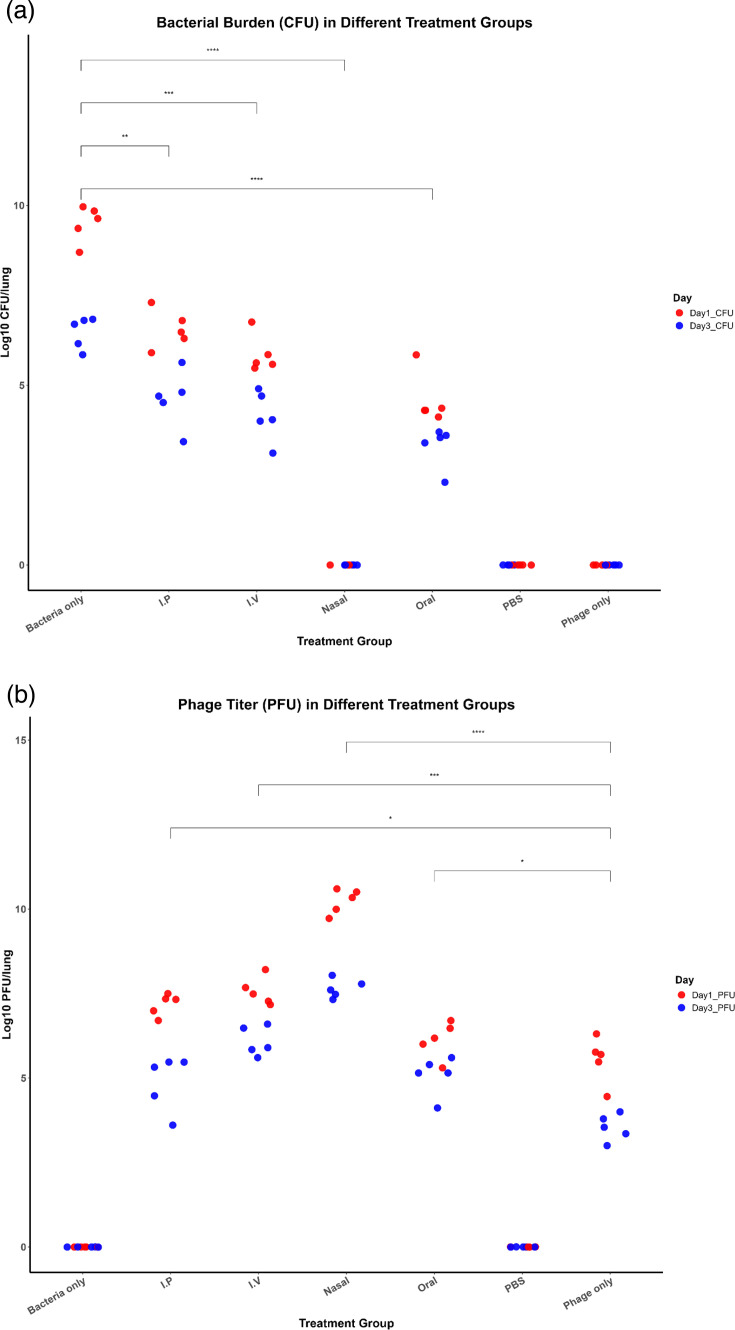
(a) Bacterial load in mouse lungs at days 1 and 3 post-infection. Comparatively reduced bacterial counts were recorded in all phage cocktail treatment groups (*P*<0.0001). (b) Phage titre in mouse lungs at 1- and 3-day post-treatments. A high phage titre indicates good therapeutic potential of the intranasal group (*P*<0.0001).

We assessed the phage cocktail’s capability to reduce bacterial load in the lungs of phage cocktail treatment groups. The study involved assessing bacterial and phage counts in the lungs of mice on days 1 and 3 post-infection as represented by Fig. 14. Each point on the graph represents a bacterial count or phage titre of an individual mouse. Mice of the positive control group exhibited elevated bacterial loads: 3.1×10^9^ c.f.u. lung^−1^ at day 1 and 2.5×10^6^ c.f.u. lung^−1^ at day 3. In contrast, the bacterial burden in groups treated with the phage cocktail decreased at both 1 dpi (day post-inoculation) and 3 dpi. Notably, intranasal administration of phages at 1 hpi resulted in complete bacterial elimination by 24 hpi, and bacterial counts remained negligible at 3 dpi. At 1- and 3 days post-infection, the orally administered phage cocktail group showed a lower bacterial burden (3×10⁴ and 2.03×10³ c.f.u. lung^−1^, respectively) compared to the intravascular group (7.2×10⁵ and 2.25×10³ c.f.u. lung^−1^, respectively). However, comparatively the i.p. phage cocktail treated group showed a higher bacterial count: 3×10^6^ c.f.u. lung^−1^ at day 1 and 3×10^4^ c.f.u. lung^−1^ at day 3. Crucially, the PBS only and phage cocktail only groups showed no detectable viable bacteria at specific time points.

The phage counts in the lungs of mice from the post-infection phage cocktail treatment group exhibited notably higher levels compared to the phage cocktail only group on days 1 and 3. Specifically, phage titres of 3.5×10^5^ p.f.u. lung^−1^ at day 1 and 3×10^3^ p.f.u. lung^−1^ at day 3 were detected in the phage cocktail only group. Notably, the nasal phage cocktail treatment group exhibited a much higher phage titre: 1.50×10^10^ p.f.u. lung^−1^ at day 1 and3.9×10^7^ p.f.u. lung^−1^ at day 3. Additionally, mice in the oral route group displayed higher phage titres compared to those in i.v. and i.p. groups. Neither the control group treated with buffer only, nor the group treated only with bacteria showed any detectable phage plaques at any time point (Fig. 14b). Based on these results, the nasal route emerged as an efficient administration method for phages, demonstrating their potential for successful therapeutic application.

### Histopathological analysis

**Fig. 15.**
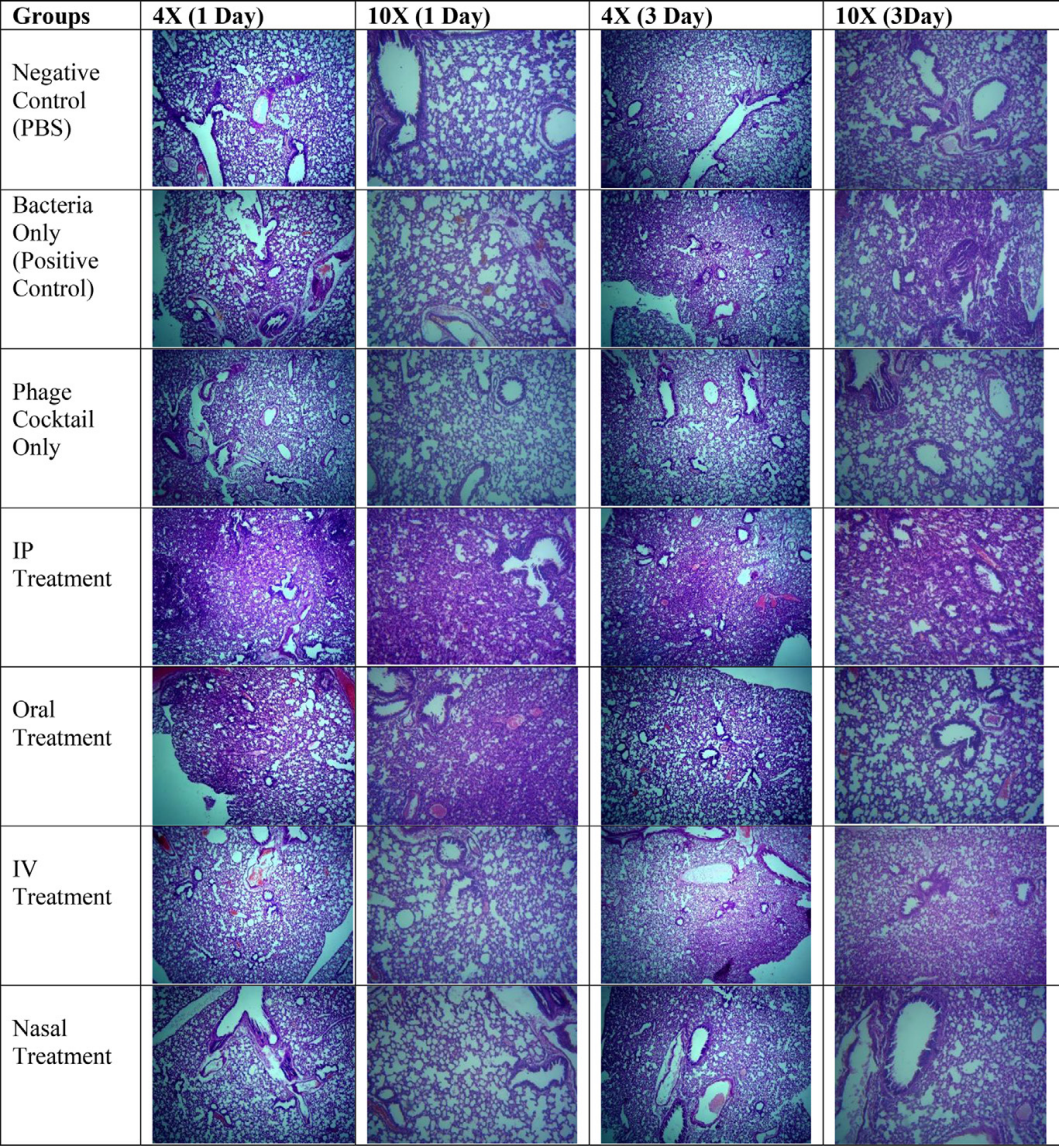
Histopathology of lung tissue on days 1 and 3 post-infection. For histological evaluation, H and E staining was used. Magnifications of ×4 and ×10 were used.

Normal lungs upon H and E-stained histological sections appear as empty spaces with thin-walled alveoli. Bronchi and bronchioles can be identified by their blue-coloured pseudostratified epithelium and pink-coloured smooth muscle layer. However, pulmonary bacterial infection alters the normal parenchymal texture and is usually manifested by leukocyte infiltration, diffuse alveolar damage, oedema, pulmonary haemorrhage and bronchiolar impaction [55, 56]. Histological results showed normal lung morphology in the PBS only and phage cocktail only groups, while the positive control group exhibited severe tissue damage and inflammation. Among treatment groups, the nasal route demonstrated the most rapid and complete recovery by day 3, followed by oral and i.v. administration. Upon histology, the nasal group exhibited complete restoration of the interstitium and patent bronchioles without any leukocytic infiltration, with day 3 lung sections closely resembling those of the PBS control group. I.p. treatment showed minimal improvement. These results are summarized in Fig. 15 and Table 3.

**Table 3.** Histopathological observations of lung tissues across different treatment groups. Good recovery shows reduced inflammation, better recovery shows marked tissue restoration, excellent recovery shows minimal inflammation and full recovery shows complete tissue restoration

Group	Day	Histological observation	Recovery status
PBS (negative control)	1 and 3	Normal alveolar structure; patent bronchi/bronchioles; no leucocyte infiltration	Normal
Bacteria only (positive control)	1 and 3	Severe leucocyte infiltration; bronchiolar impaction; interstitial expansion	Severely infected
Phage cocktail only	1 and 3	No histological changes observed	Normal
I.p. treatment	1	Heavy leucocyte infiltration; bronchiolar impaction; patchy haemorrhage	Poor
	3	Slight improvement; persistent inflammation	Slow recovery
Oral treatment	1	Mild inflammation	Moderate
	3	Restoration of alveolar spaces; patent bronchioles	Better recovery
I.v. treatment	1	Leucocyte infiltration; haemorrhagic foci; patent bronchioles	Moderate
	3	Reduced inflammation; better than i.p.	Good recovery
Nasal treatment	1	Minimal leucocyte infiltration; restored bronchiolar patency	Excellent
	3	Comparable to PBS group; complete tissue restoration	Full recovery

## Discussion

Our current study is focused on investigating the therapeutic potential of two newly isolated *A. baumannii* phages Srynta and Nolivar from sewage water, which belong to the class Caudoviricetes*. In vitro* characterization revealed their good stability across different conditions, as well as their efficient adsorption, effective lysis of host cells and short latent period and sufficient burst size. Both phages lysed five out of six *A. baumannii* strains tested at varying e.o.p. values, which is higher than phage AB1, which infected only one of five strains reported by Yang *et al*. [57]. In our study, both phages Srynta and Nolivar demonstrated high thermal stability with only a 2.6–3.5 log_10_ p.f.u. ml^−1^ reduction after 1 hour of incubation at 60 °C. These results contrast with those reported by Bagińska *et al*. [58], where phages Acba_6, Aclw_9 and Acba_11 exhibited more pronounced declines in stability, with titre reductions of ~4.4, 3.5 and 3.2 log_10_, respectively [58]. This suggests that Srynta and Nolivar may possess comparatively higher thermal tolerance, potentially broadening their applicability under varying environmental and therapeutic conditions. In our study, both Srynta and Nolivar phages exhibited a high adsorption rate of 95% within 10 min, which is comparable or slightly higher than reported by Leshkasheli *et al*. [59] where *A. baumannii* phages vB_AbaM_3090 and vB_AbaM_3054 showed an adsorption rate of 95±2% and 89±4% after 10 min [59]. In comparison, a previous study by Baginska *et al*. [60] reported an 82% adsorption rate for *Acinetobacter* phage Acba_6 at a higher m.o.i. of 0.1 [60]. Both phages demonstrated sufficient host lytic activity at all m.o.i. values (10, 1 and 0.1) as previously reported for other *Acinetobacter* phages [61]. In contrast to *Acinetobacter* phage vB-AbaS- Loki, which exhibited a 40 min latent period and a burst size of 43 p.f.u. infected cell^−1^, the phages Srynta and Nolivar demonstrated a shorter latent period of 30 min and higher burst sizes of 100 and 81 p.f.u. infected cell^−1^, respectively [62].

Both Srynta and Nolivar phages have linear dsDNA and have genome sizes of 45,218 bp and 41,230 bp which are comparable to other myovirus phage genomes like 45,415 bp and 42,654 bp of *Acinetobacter* phage WCHABP12 and Acibel007, respectively [63, 64]. Total G+C content of Srynta (38 mol%) and Nolivar (37.9 mol%) phages is in accordance with other *Obolenskvirus* phages like vB_AbaM_AB3P2 (37.75%), Brutus (37.40%) and Scipio (37.60) [65, 66]. As the majority of encoded proteins were hypothetical, only 39 and 36 proteins with known functions were predicted in the genomes of Srynta and Nolivar phages, respectively. Phylogenetic analysis and ANI comparisons indicated that Srynta and Nolivar phages were closely related, representing two distinct strains within the genus *Obolenskvirus,* and both exhibit characteristic myovirus morphology. Genomic analysis confirmed that antimicrobial resistance, bacterial virulent factors and lysogeny genes were not present in both phages. These attributes suggested them as strong candidates for *in vivo* phage treatment trials.

To ensure the potential clinical applicability of phage therapy, a comprehensive understanding of phage biology and *in vivo* efficacy is essential. With increasing clinical trials demonstrating the safety and efficacy of phage therapy, including cases of severe infections, the promise of bacteriophage-based therapeutics is evident [67, 68]. This study advances current efforts in phage therapy by presenting the detailed characterization and therapeutic effects of Srynta and Nolivar phages in a mouse lung infection model of *Acinetobacter b. 3–695*. The current 3 week study of the mouse model showed 100% survival of animals by treating intranasally with phage cocktail at m.o.i. 10 at 1 hpi, which is comparatively higher than the 83.33% survival rate of the 2-week study of Hua *et al*. (2018) [11]. In our study, survival rates of 83% at m.o.i. 1 and 66% at m.o.i. 0.1 are comparatively higher than those reported by Jeon *et al.* (2019), which were 60 and 30%, respectively [12]. In another study of Wang *et al*. [69], a survival rate of 70 and 20% at m.o.i. 1 and m.o.i. 0.1 was observed, which is comparatively less than reported in the present study.

In our study, four different phage administration routes were used to evaluate phage therapy *in vivo* using a phage cocktail after 1 h bacterial challenge. Survival comparison showed that intranasal and oral administration of phages were beneficial and reduced the severity of the disease. In a previous study of Cha *et al*. [6], a cocktail of five phages at m.o.i. 10 was administered intranasally at 4 hpi and a 35% survival rate was observed, which is lower than reported in our study. In the i.p. and i.v. groups, higher bacterial counts and reduced phage titres were observed. These findings are consistent with previous studies showing that phages administered intraperitoneally or intravenously were rapidly eliminated from the blood and organs of mice, resulting in poor protection [70]. Our results showed that intranasal phage therapy is a suitable method for the treatment of lung diseases, given the high phage titres in the target organ. More interestingly, in the phage cocktail only group, there was a significant phage titre of 3.5×10^5^ p.f.u. lung^−1^ was recorded at 24 h after inoculation. Importantly, even without an active infection, the phage cocktail persisted in the lungs for an extended duration, highlighting its stability and potential as a prophylactic agent [71]. This was unexpected as many studies reported rapid clearance of phages from the body [72].

Results of histopathological analysis indicate that the intranasal administration of the phage cocktail effectively treats infection caused by CRAB strain 3–695 without inducing adverse effects. The i.p. group showed the slowest response with day 1 showing worsening of disease with heavy infiltration of leucocytes, impaction of bronchioles, expansion of interstitium and focal haemorrhages. Oral group response was better than i.v. group and i.v. was better than i.p. group. The strong efficacy of the oral route could be attributed to the systemic translocation of phages from the gastrointestinal tract to the site of infection in the lungs.

In conclusion, phages Srynta and Nolivar have been thoroughly characterized and demonstrate potent therapeutic effects against CRAB strain 3–695 infection in a mouse model. This study contributes to the growing body of evidence supporting the use of phages as alternative therapeutic agents against MDR bacterial infections. With their high specificity and safety profile, phages hold promise for combating the challenges posed by antibiotic-resistant pathogens.

## Abbreviations

ANI: average nucleotide identity
CDS: coding sequence
CLSI: Clinical & Laboratory Standards Institute
CRAB: Carbapenem-resistant *Acinetobacter baumannii*
dpi: day post-inoculation
EOP: Efficiency of plating
hpi: hours post-infection
i.p.: intraperitoneal
i.v.: intravenous
MDR: multidrug-resistant
OD: Optical density
PBS: Phosphate buffered saline
TEM: transmission electron microscopy
VICTOR: Virus Classification and Tree Building Online Resource
